# Shedding Light on Degradation Gradients in Celluloid: An ATR-FTIR Study of Artificially and Naturally Aged Specimens

**DOI:** 10.3390/polym15030522

**Published:** 2023-01-19

**Authors:** Marco Valente Chavez Lozano, Christina Elsässer, Eva Mariasole Angelin, Marisa Pamplona

**Affiliations:** Conservation Science Department, Deutsches Museum, Museumsinsel 1, 80538 Munich, Germany

**Keywords:** artificial aging, cellulose nitrate, nitrogen content, carbonyl groups, cultural heritage, historical plastics, attenuated total reflectance Fourier transform infrared spectroscopy (ATR-FTIR), degradation gradients

## Abstract

Celluloid artifacts are known by conservation professionals to be prone to degradation, threatening their own integrity and that of nearby heritage collections. Celluloid alteration can have a heterogeneous nature, and this research topic is still in its infancy for heritage science. This article investigates degradation gradients, both along depth and width, of artificially aged celluloid sheets, and compares them to three-dimensional (3D) historical objects with the aim of gaining a better insight into the nature and evolution of their decay. ATR-FTIR was used to systematically study different sampling points of the artificially and naturally aged specimens and allowed us to recognize better-preserved surfaces and more deteriorated cores. ATR-FTIR was found suitable for assessing the molecular changes induced by degradation, particularly denitration and formation of carbonyl-containing degradation products in severely aged specimens. Even though the severely artificially aged sheets displayed unusual alteration phenomena, they present a degradation gradient similar to the one observed for the naturally aged 3D objects under study. This research underlines that sampling at different depths and/or widths is relevant for characterizing the heterogeneity of degraded celluloid, and further investigation with chromatographic techniques would greatly benefit the understanding of the complex degradation of celluloid artifacts.

## 1. Introduction

Cellulose nitrate (CN) is the cellulose derivative resulting from the substitution of side hydroxyl groups (OH) with nitro (NO_2_) groups. For the total weight of a CN chain, the mass percentage attributable to nitrogen (N) would be equal to 14.14%, 11.1% or 10.5% if, respectively, all three, two or only one of the hydroxyl groups per monomeric unit would have been substituted with nitro groups [1,2]. The mixture of moderately nitrated CN (nitrogen mass content ≤12% [3,4]) with up to 33% weight camphor as plasticizer (Figure S1) is known as celluloid [5].

Celluloid was employed for the industrial production of transparent films, sheets and three-dimensional (3D) artifacts. It could also be mixed with appropriate fillers or pigments to imitate expensive materials, such as ivory, mother-of-pearl and tortoiseshell in jewelry cases, brooches, dolls, etc.

Celluloid artifacts are widely spread in archives, cinematheque and modern museum collections. These objects are well-known by conservation professionals for being highly sensitive to degradation [5,6]. Yellowing, embrittlement, loss of plasticizer and emission of volatiles are typical signs of celluloid alteration. Aside from flammability problems, their high chemical instability may lead to the total collapse of the object itself and poses a threat towards surrounding assets in the collection.

As a consequence, efforts have been made to study celluloid degradation. As it would not be practical to wait for several years to observe their natural aging, artificial aging protocols, including CN and celluloid samples, have been adopted to simulate their natural degradation in shorter times. Past artificial aging experiments studied the thermal stability of highly nitrated cellulose by subjecting it to high-temperature conditions (100 °C–200 °C) [7]; or exposing it to ignition conditions [8]. However, the resulting kinetics of degradation under such conditions are not necessarily extrapolatable to the aging occurring at lower temperatures and could not serve for predicting long-term natural aging [1]. Thermal degradation studies at relatively low-temperature ranges (T < 100 °C) have been performed by Trache and Tarchoun [3], Chin et al. 2007 [9] and Shashoua et al. [10].

Most recent studies have preferred instead photodegradation, especially under UV-visible light filtering wavelengths below 300 nm, to represent outdoor weathering due to the faster rate of degradation obtained when compared to pure thermal aging at low temperatures [11]. These studies include the work by Neves et al. [11,12], Bussiere et al. [4], Berthumeyrie et al. [13] as well as Hon and Gui [14].

Few artificial aging studies have employed humidity coupled with high temperature. These include early works on the aging of cinematographic films with CN bases [15,16] and the research by Quye et al. [17], which employed a temperature of 70 °C and different relative humidity (RH) conditions, going up to 75%.

Besides the sublimation of camphor, the loss of nitro groups and the creation of carbonyl (C=O) functions can be typically linked to the two main pathways that rule the degradation of celluloid with moderate N mass percent content when the temperature remains below 100 °C and illumination is kept above 300 nm [11,12,18].

**Figure 1:**
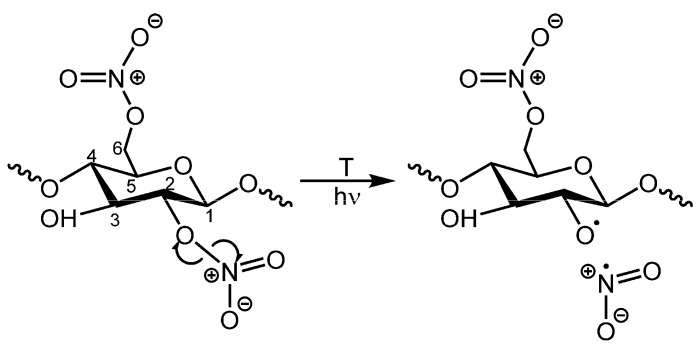
Scheme illustrating the initial homolysis of NO_2_ groups in CN [7]. NO_2_ groups can be attached to C2, C3 and C6 positions.

The first pathway begins with the homolytic scission (homolysis) of RO-NO_2_ bonds (Figure 1), a reaction with a low activation energy (24–26 kcal/mol [19]), predicted to occur at a very low rate at ambient temperature [3,20]. This homolytic scission can be caused by thermal or photo-excitation processes. Photooxidation has been proposed to follow the same chemical pathways as thermal degradation [11,12,18] but at a much faster rate [21].

Nitrogen dioxide radicals (^•^NO_2_) and alkoxy macro radicals (RO^•^) [19,22] result from the homolytic scission reaction (1). These radicals have been held partially responsible for the autocatalytic nature of CN degradation, as they can undergo further complex oxidation reactions even at room temperature [3,21]. The C2 position on the cellulose ring (Figure 1) is the most labile because it presents the weakest bond at 39.89 kcal/mol (equivalent bond in C6 has 78.82 kcal/mol bond energy) [22,23]. Reaction (1) accelerates with the length of the hydrocarbon chain and when the N mass surpasses 4% due to the influence of adjacent nitro groups, which are strongly electronegative [19].

RONO_2_ → RO^•^ + ^•^NO_2_ (1)

From this stage onwards, several radical reactions can take place at the different C positions [11,12,13]. If H abstraction occurs at C3 position after denitration, it leads to the formation of a cyclic ketone containing a C=O function. If H abstraction occurs instead at C1 or C5, alkoxy and hydroperoxide radicals are formed, leading both to denitration and to continuous chain scission by the formation of gluconolactone intermediates and ending with the formation of an anhydride [11,13].

Summing up, photo-thermal degradation reactions lead to the complete denitration of the cellulose ring, to the creation of C=O functions and to the decreasing molecular weight of the CN chain.

**Figure 2:**
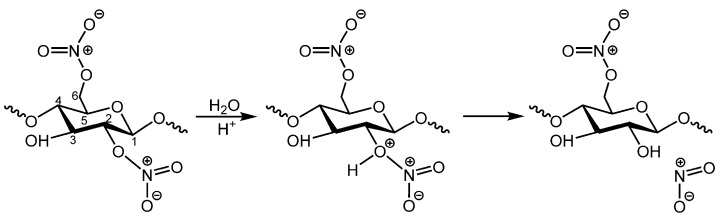
Scheme illustrating the most probable pathway of hydrolysis of the ester links when it compromises the nitro groups [25].

The second degradation pathway of celluloid involves the hydrolysis of ester links [1,22]. This may compromise nitro groups (Figure 2) or glycosidic bonds in the main CN chain following the pathway observed during acid treatment of pure cellulose, which is easier to occur in the amorphous regions of cellulosic materials [24]. It has been suggested that CN hydrolysis, when it compromises the nitro groups, can occur even in neutral conditions in the presence of humidity. However, recent computational calculations with simplified models suggest that the process is only likely to occur in acidic conditions for moderately nitrated CN [25]. Hydrolytic denitration leads to the formation of alcohol (ROH) on the cellulose ring, accompanied by the emission of nitrogen oxide gasses (NO_x_). The reaction of highly reactive nitrogen dioxide (NO_2_) with moisture in the air forms nitric acid (HNO_3_) [1,9,19].

It is important to notice that both nitrous and nitric acids (HNO_2_ and HNO_3_) are among the most dangerous products which can be formed from ^•^NO_2_ radicals, either by ^•^H or reaction with water [11,12,13]. These acids are highly oxidizing and cause a decrease in pH, which in turn can trigger acid hydrolysis or speed up acid formation [18]. Acid-induced hydrolysis occurs rapidly due to its low activation energy (19.1–26.3 kcal/mol [2]), which is lower than the one required for thermal NO_2_ detachment [19]. As photo-thermal degradation, hydrolysis can also lead to the formation of degradation products with C=O functions.

Artificial aging studies are useful not only to understand the degradation of celluloid but also to test the effectiveness of conservation treatments, aiming to help heritage institutions to implement the best storage and exhibition practices. In this framework, a recent study conducted at the Deutsches Museum aimed at assessing the effect of low storage temperatures on the conservation of 3D celluloid artifacts [26,27,28]. In the initial stage, the project aimed at producing celluloid sheets in moderate (i.e., showing only subtle visual changes but presenting off-gassing) and severe (i.e., significantly altered and presenting off-gassing) degradation conditions. For that purpose, celluloid sheets were artificially aged for 10 and 13 days, respectively, at 70 °C and 75% RH. However, the first set of sheets reached a condition accurately representing moderately degraded naturally aged objects; the sheets aged at 13 days displayed unusual phenomena. They did however present a degradation gradient along depth similar to the one observed in severely degraded celluloid, i.e., the outer surface was in better condition than the degraded core. Even though the gradient along depth is reported to be typical for severely degraded 3D celluloid objects in collections [23,29,30,31], degradation gradients along width can also occur [27]. However, no studies are known to the authors presenting a systematic approach using artificially aged specimens to describe these phenomena in 3D objects.

As such, this work aims, in the first instance, to assess molecular changes and related gradients induced by artificial aging after 10 and 13 days in order to characterize their nature and evolution over time, in width and depth. Secondly, the molecular changes detected in the 13-day aged sheets were compared with those of naturally aged objects, to determine to which extent their degradation gradient is alike.

Fourier transform infrared spectroscopy (FTIR) has been used for the investigation of celluloid historical objects and artificial aging experiments in different acquisition modes [4,17,18,21,27,32]. Therefore, attenuated total reflectance (ATR) was selected for this study due to its high reliability, ease of interpretation and lack of any sample preparation. As the loss of camphor, denitration and formation of degradation products containing C=O functions are considered the main decay markers of celluloid [3,18,33,34], their changes were assessed by a semi-quantitative approach.

## 2. Materials and Methods

### 2.1. Reference Materials

Racemic camphor 96% (CAS 76-22-2, Thermo Electron) and CN membranes (GE Healthcare) were used as reference materials to characterize the main components constituting celluloid.

### 2.2. Celluloid

Transparent celluloid industrially produced by Rothko and Frost™ (Incudo Clear Transparent Celluloid Sheet IN2322, 430 × 290 × 1 mm) was used and saw-cut into square sheets (4 × 4 cm). A hole was drilled in one corner of each sheet so they could be hung on a glass rod to ensure good air circulation during aging.

### 2.3. Artificial Aging of Celluloid Sheets

**Figure 3:**
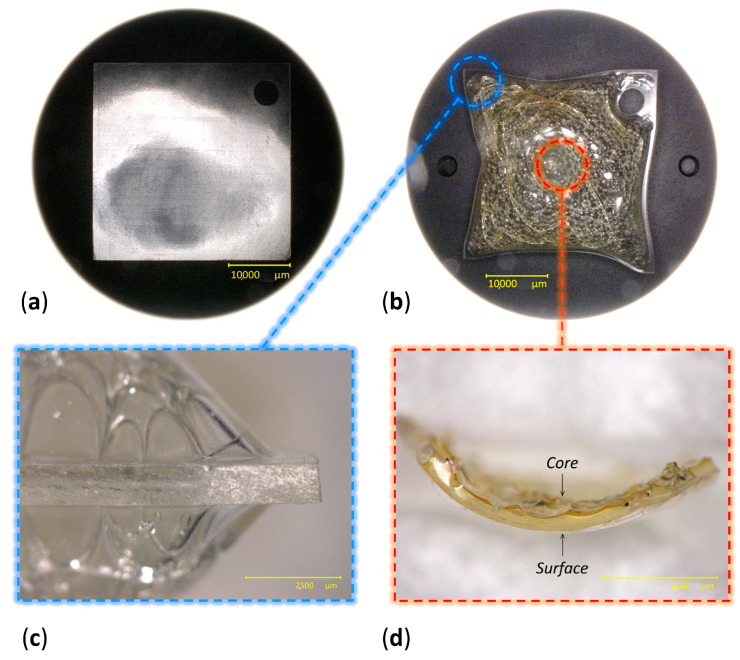
Views of celluloid sheets at 0 days (**a**) and after 13 days of aging (**b**). Details of the lateral view of a 13-day aged sheet (**c**) and of the depth-related gradient of degradation observed at the center of a sheet (**d**).

In total, 80 Celluloid sheets were aged at 70 °C and 75% RH in a fan-assisted dynamic climate chamber (MKF 115, Binder), ensuring good aeration of the samples by hanging them on glass rods. After 10 days, 40 sheets achieved a moderate condition of being slightly yellowed. After 13 days, 40 sheets showed severe discoloration and physical alteration (Figure 3). In particular, bubbles, crazes and fractures were observed in the central area of the sheets, while no visual changes were visible along the borders.

After aging, both aged and unaged sheets were kept in the dark at room temperature inside a safety storage cabinet (Q90.195.120, asecos^®^) with permanent filtered (active charcoal) air ventilation.

**Figure 4:**
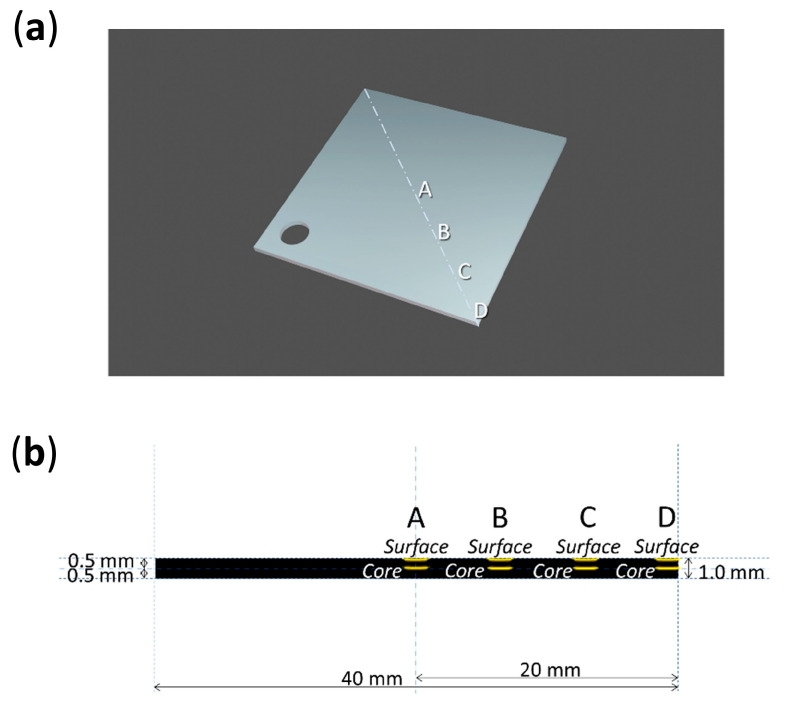
Sampling points (**a**) and depths (**b**) of 0-day and 10-day aged sheets.

**Figure 5:**
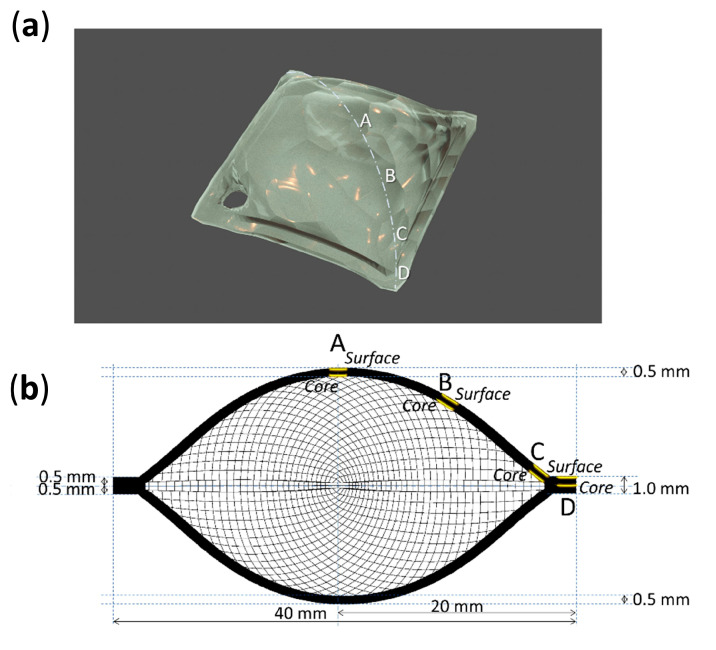
Sampling points (**a**) and depths (**b**) of 13-day aged sheets.

Four analysis points were considered along the diagonal of each sheet, from its center (A) to its corner (D) (Figure 4 and Figure 5). Micro-samples with a thickness of ca. 100–200 µm for each point were taken with a surgical scalpel at the surface and at the core (at a depth of ca. 500 µm). Sampling was performed under a stereo microscope, and depth reproducibility was ensured by documenting the sheets’ section before and after sampling with a Keyence VHX-1000 digital microscope under a 5–50x magnification.

### 2.4. Naturally Aged Objects

**Figure 6:**
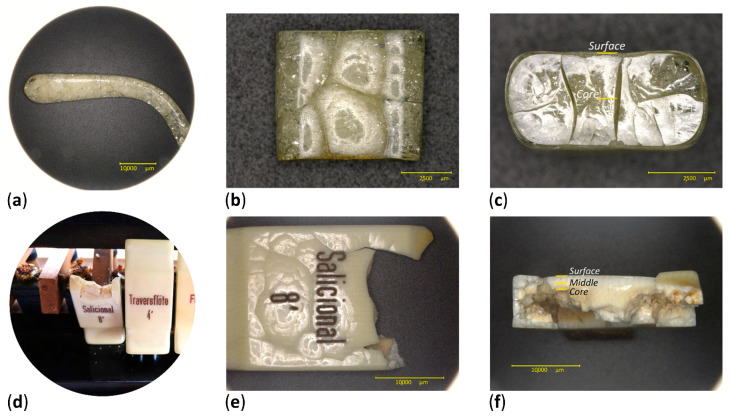
Original temple (**a**); side (**b**) and section (**c**) views of the analyzed fragment. Original condition of the organ key before being removed from the instrument (**d**); detail from the front (**e**) and section (**f**) views of the organ key. Sampling points are indicated on the sections of both objects.

Two historical artifacts were selected. The first was a transparent eyeglass temple (Figure 6a–c), formally part of the Deutsches Museum optic’s collection, which had been removed from the inventory due to its advanced degree of alteration. The surface of the eyeglass temple appeared transparent and continuous, whereas its core seemed cracked and brittle. The second case study was a white-colored organ key, previously belonging to a musical instrument from a Bavarian church (Figure 6d–f). The surface of the organ key remained white and coherent, whereas its core appeared discolored, cracked and brittle. The fillers in the organ key were previously identified as titanium oxide and zinc oxide by means of Raman and X-ray fluorescence spectroscopies.

As both historical objects appeared to retain a homogeneous condition along their moderately damaged surfaces, sampling was performed only considering their section. Two micro-samples were taken from the eyeglass temple, one at the surface and another at the core. Since the organ key was thicker, three sampling depths were chosen along its section: at the surface, middle and core, following the gradient of visual degradation.

### 2.5. Attenuated Total Reflectance Fourier Transform Infrared Spectroscopy (ATR-FTIR)

The ATR-FTIR spectra were acquired with an Alpha FTIR Spectrometer (Bruker) equipped with a diamond ATR crystal controlled through the OPUS 8.1 software (Bruker). The spectra were collected by integrating 64 scans for each measurement in the 4000 to 400 cm^−1^ range with a 4 cm^−1^ spectral resolution. At least two spectra were acquired from each micro-sample. Enough material was collected from the historical objects to allow the acquisition of at least three spectra per micro-sample.

**Figure 7:**
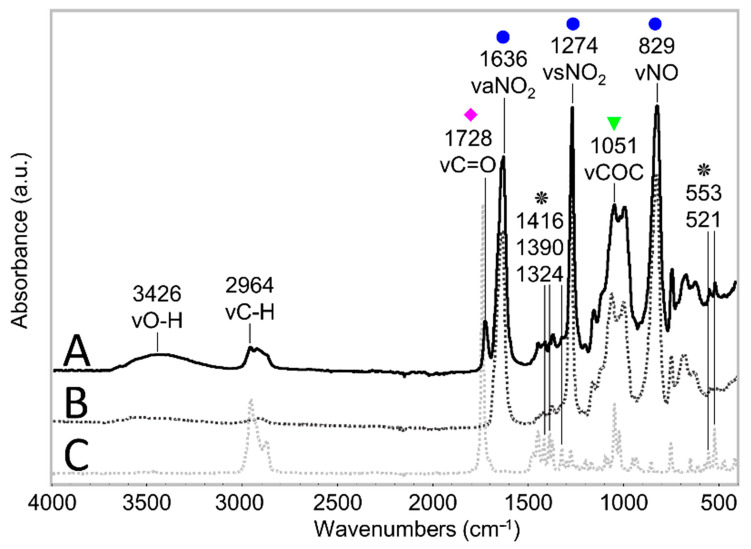
ATR-FTIR spectra of an unaged celluloid sheet (A), of pure CN (B) and camphor (C) references. The COC stretching reference band is highlighted with a green triangle, probe bands related to the NO_2_ group are marked with a blue circle, and the C=O stretching band is highlighted with a magenta diamond. Bands representative of camphor presence are marked with a black star. Each spectrum has been normalized considering the strongest infrared band.

The ATR-FTIR spectra of the two components of celluloid, cellulose nitrate (CN) and camphor, are depicted in Figure 7. Full band assignments are reported in Table 1.

Three sheets of each aging time were randomly selected for conducting the analyses because they showed similar conditions among each other.

The absorbance of each probe band was measured using the peak height tool of the OMNIC 7.2 software (Thermo Electron Corporation) without any correction or further manipulation of the spectra. An average ratio was calculated between the absorbance of each probe band (νC=O at 1730 cm^−1^, ν_a_NO_2_ at 1634 cm^−1^, ν_s_NO_2_ at 1273 cm^−1^ and νNO at 826 cm^−1^) against the absorbance of the reference band (νCOC at 1053 cm^−1^) [18]. The absorbance calculations were performed at the maximum for each band in every individual spectrum, drawing for each band a baseline always using the same points: from 1840 to 1503 cm^−1^ for ν_a_NO_2_ and νC=O bands; from 1503 to 1187 cm^−1^ for ν_s_NO_2_ band; from 1187 to 930 cm^−1^ for νCOC band; and from 1189 to 771 cm^−1^ for νNO band. In the spectra of the eyeglass temple and organ key, the baselines were drawn differently: from 1187 to 881 cm^−1^ for the νCOC band and from 1187 to 785 cm^−1^ for νNO band because they showed significant variations that required tailored intervals for detecting the peak height. The standard deviation (SD) among original measurements was used to assess the statistical difference between the obtained averaged values.

## 3. Results and Discussion

**Figure 8:**
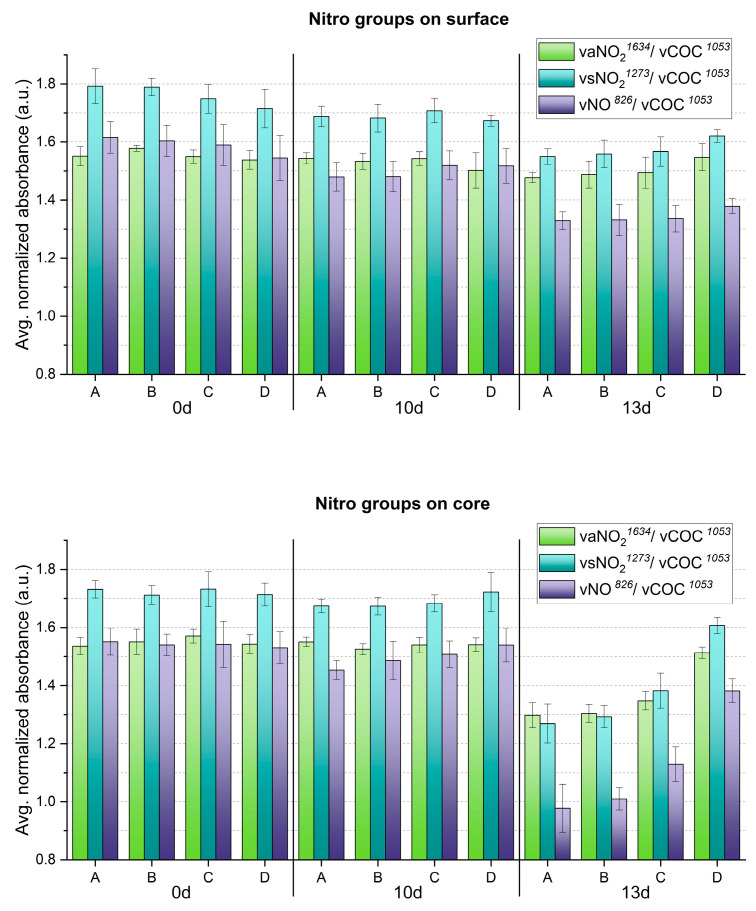
Averaged and normalized ATR-FTIR absorbance for NO_2_ groups of the artificially aged celluloid sheets. A, B, C and D refer to sampling points. Error bars correspond to the SD.

**Figure 9:**
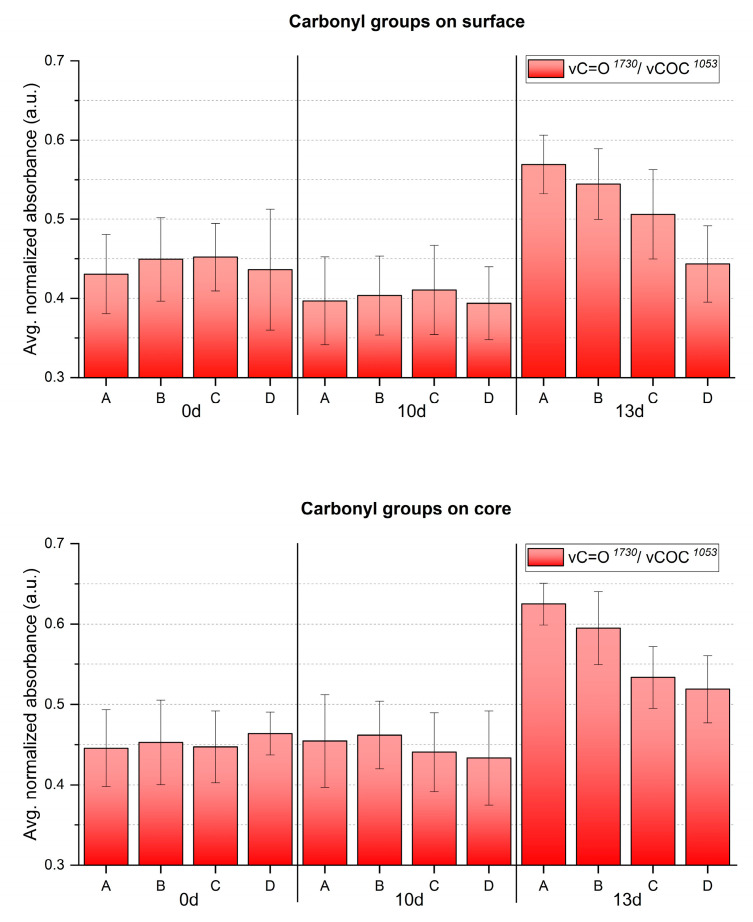
Averaged and normalized ATR-FTIR absorbance for C=O groups of the artificially aged celluloid sheets. A, B, C and D refer to sampling points. Error bars correspond to the SD.

The evolution of molecular changes and related gradients along both depth and width at different aging times was successfully characterized by ATR-FTIR (Figure 8 and Figure 9). Generally, the nitro groups were observed to decrease, particularly at the central area of the sheets, with the first evidence already after 10 days. Instead, a clear opposite trend was detected for the νC=O probe band at 13 days of aging. In general, the evolution of the bands ν_s_NO_2_ at 1273 cm^−1^ and νNO at 826 cm^−1^ was more descriptive in registering molecular variations with aging than the ν_a_NO_2_ signal at 1634 cm^−1^.

In accordance with visually evident alteration, ATR-FTIR detected statistically significant modifications between surface and core after 13 days, identifying a higher degree of denitration at the core. A gradient along width was also observed from the border (point D), showing higher signals of the nitro groups, towards the center (point A) with lower nitro group absorption. The carbonyl group increased significantly after 13 days, highlighting the greatest molecular change at the core, even for the border areas (point D), not affected by severe visual degradation. Just as for the nitro groups, a gradient of carbonyl signal intensity along the sheet’s width was detected, being more intense at sampling point A than at sampling point D, in correspondence with the severity of degradation. In summary, the analytical evidence correlates well, i.e., higher denitration along a more intense signal of carbonyl groups, highlighting the severest extent of degradation at the core of the sheet center (point A) after 13 days.

The mechanisms responsible for denitration can be both thermolytic and hydrolytic, due to the simultaneous presence of high temperature and humidity, influencing one another and giving rise to fast, complex and intertwined degradation reactions. For example, ^•^NO_2_ is formed by initial thermolysis, and by H abstraction at the RH groups of CN, it gives rise to an R^•^ radical, which leads to further oxidation and the creation of HNO_2_, which can trigger acid hydrolysis. This reaction (2) is enhanced by the relatively low solubility of NO_2_ in CN [19].

RH + ^•^NO_2_ → R^•^ + HNO_2_ (2)

It is possible that hydrolysis of CN occurred at an early stage because a certain amount of water was already adsorbed by the celluloid sheets due to CN’s slight hygroscopicity [1,19] and relatively high permeability coefficient towards water (4.72 Pa^−1^ [41,42]) at room temperature. Additionally, Manelis et al. state that below 70 °C, a humidity of 1% (easily adsorbed from the surrounding air) induces hydrolysis at a higher rate than primary NO_2_ detachment due to the presence of free OH groups [19], further reinforcing the notion that hydrolysis played an important part since the beginning. Therefore, one can infer that the high humidity conditions at 75% RH at 70 °C could greatly promote hydrolysis of the nitro group.

As mentioned in the Introduction, thermal degradation leads to the creation of cyclic ketones, gluconolactones and anhydrides as carbonyl-containing degradation products, but hydrolytic degradation has been suggested to also give rise to the formation of aldehyde (RCHO) functions (3), containing C=O groups, upon hydrolysis of the NO_2_ group at the C6 position [1], or by reaction of nitric acid with ROH [9,43] (4).

RCH_2_ONO_2_ + OH^−^ → RCHO + H_2_O +NO_2_^−^ (3)

ROH + HNO_3_ → RCHO + HNO_2_ + H_2_O (4)

The formation of degradation products containing carbonyl groups during artificial aging of CN has been previously reported by UV spectroscopy [14], synchrotron deep UV photoluminescence micro spectral-imaging [11] and analysis after chemical derivatization which, all together, explain the influence of carbonyl-containing degradation products in the νC=O IR band [13]. This was evidenced in the FTIR spectra of photo- and thermally aged pure CN samples by a clear broadening and increase in intensity in the carbonyl region in 1730–1740 cm^−1^ [4,15]. However, the intensity of νC=O absorption due to aging in celluloid is contemporarily influenced by two variables [35]: the loss of camphor that induces its decrease, and the formation of carbonyl-containing degradation products that leads to the bands` increase. Both νC=O functions of camphor and carbonyl-containing decay products present IR bands in the same spectral region [4,11,12,35]. Thus, as aging progressed, the νC=O absorption of celluloid samples in the present research (Figure 9) cannot be solely related to the camphor content and it is not suitable to estimate the camphor loss due to aging. Indeed, other representative bands of camphor should be considered (marked with a black star in Figure 7). In the present investigation, a very small decrease in their relative intensity after 13 days of aging was observed, which suggests that the increase in the νC=O absorbance is likely mainly attributable to the creation of degradation products containing carbonyl groups.

After 13 days, the artificially aged celluloid sheets displayed unexpected degradation effects. To pose hypotheses about the mechanisms that might have induced such degradation, it was necessary to correlate infrared data with visual appearance, considering the sampling points.

The volume expansion at the center of the sheets could be related to the formation of volatiles, which would remain trapped and induce internal pressure. The accumulation of volatiles in the bulk of celluloid has already been reported to occur in naturally aged 3D objects [5]. Such expansion was not observed at the borders of the sheets, partly because their increased surface area would likely facilitate the aeration and diffusion of the emitted gasses and perhaps due to structural differences of the CN chains in this region induced by the heat during saw-cutting, altering its porosity and therefore its permeability [25]. In the authors’ opinion, extreme volume expansion is unlikely to occur for thin celluloid films, which would enable a faster and easier diffusion of volatiles than thicker objects, as reported by Quye et al. [17]. Another factor that should be considered is that the employed celluloid sheets appear to have been fabricated by blending two thinner sheets of 0.5 mm each (Figure 5). This supposition is reinforced by the observation that all sheets showed a symmetrical volume deformation as if the two layers would have been detached along their joint. This interface (not yet recognizable at 0 days) became apparent at the borders of sheets after 13 days of aging (Figure S2).

The development of brown color in the core can be attributed to the accumulation of NO_x_ species [3] and to N_2_O_4_ in particular due to the reddish-brown discoloration [9] (Figure 3d), as nitrous oxide gases have been reported to be lowly soluble in CN [19]. The developed gases may very likely promote further denitration and hydrolysis autocatalytically.

When collecting micro-samples from the 13-day aged sheets, it was noticed that the core was rigid while the surface was still flexible. This rigidity can be related to a higher crystallinity due to the formation of OH functions at the expense of NO_2_- groups, which would incite the formation of intramolecular and intermolecular hydrogen bonds [3].

**Figure 10:**
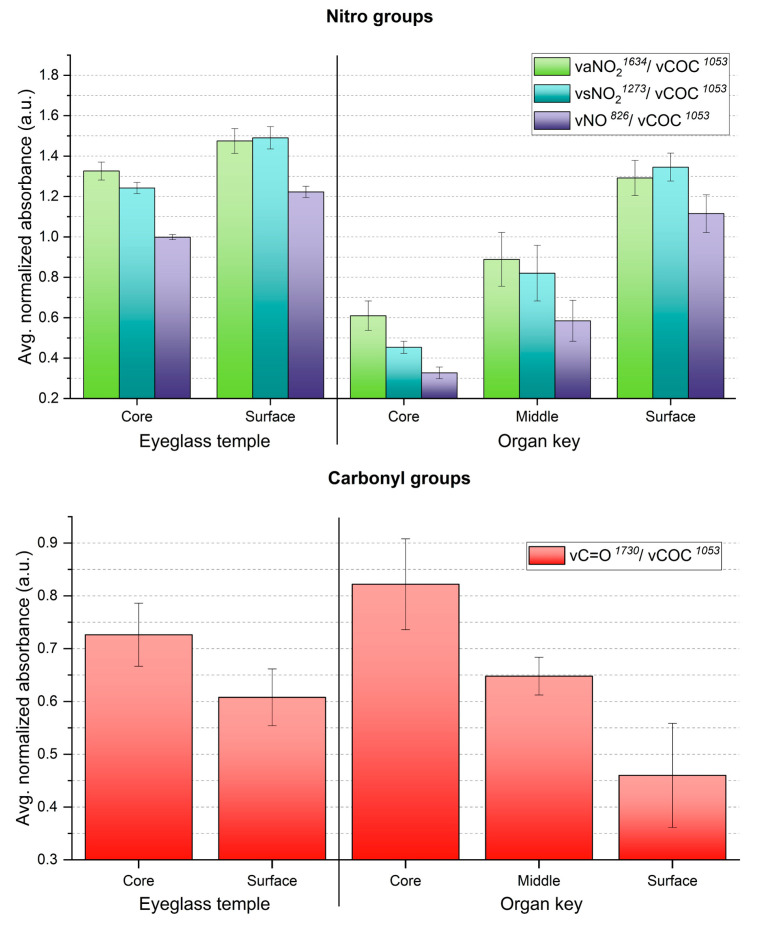
Averaged ATR-FTIR absorbances for NO_2_ (**up**) and C=O (**down**) groups of the two naturally aged CN objects. Error bars correspond to the SD.

ATR-FTIR analysis also showed that the gradients of the 13-day aged sheets and two historical celluloid objects are similar in what concerns the trends of NO_2_ and C=O groups (Figure 10). Indeed, the core of the eyeglass temple and organ key displayed the severest extent of degradation, as measured for the 13-day aged sheets. The trend of the ATR-FTIR absorbance ratios of both historical objects at the different sampling points matches with their degradation phenomena in section (Figure 6c,f). Interestingly, both 13-day aged sheets and organ key display severe discoloration in their core (Figure 3d and Figure 6f).

The formation of NO_x_ gasses, as a consequence of the loss of the nitro groups, likely occurred in 13-day-aged celluloid sheets and historical objects. However, bubbles were observed in the artificially aged sheets. The high temperature and high humidity in the aging chamber could have softened the CN in celluloid, which is a common behavior of thermoplastic polymers [44,45,46]. The celluloid, in turn, could have been easily deformed due to the pressure of the gas formation. To the best of the authors’ knowledge, bubbles have never been observed in 3D historical objects. The chances of having the material be deformable at room temperature are much less, and the material would likely release stresses by forming cracks, which is more typical for celluloid degradation.

**Figure 11:**
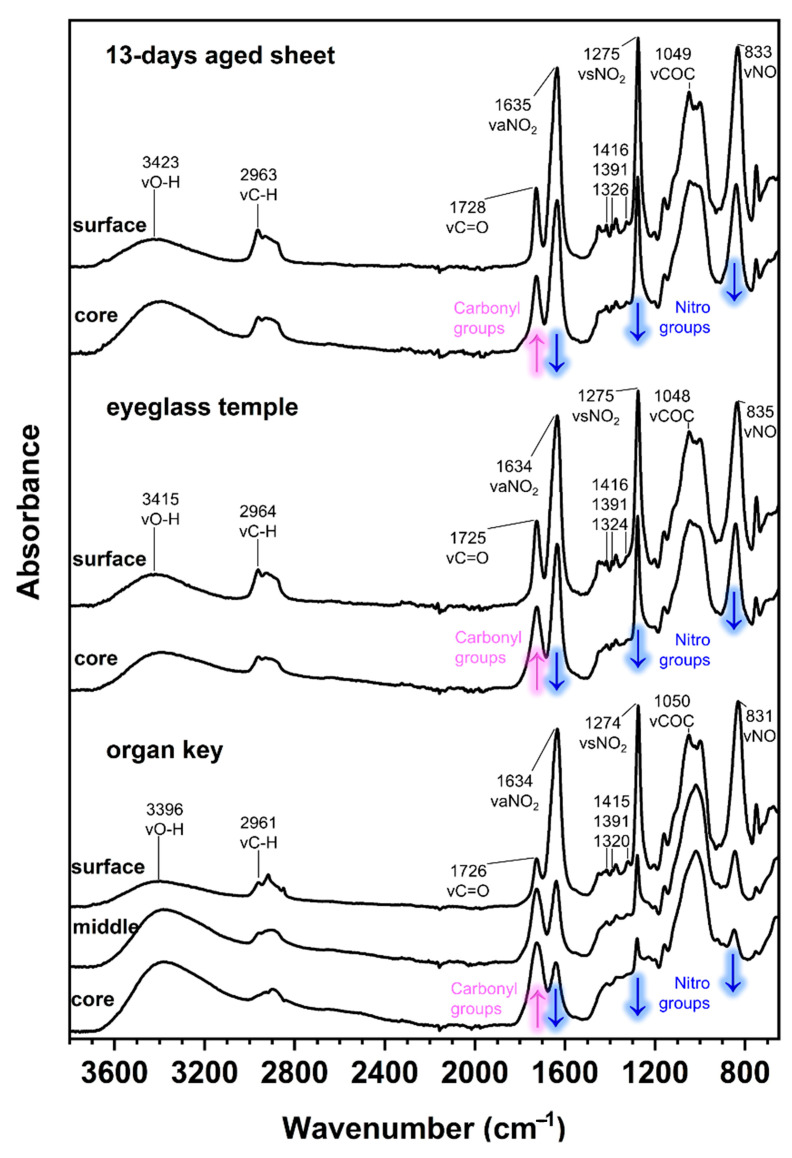
Normalized ATR-FTIR spectra of the 13-day artificially aged sheets collected in position A (**up**), eyeglasses temple (**middle**) and organ key (**bottom**) at different depths. The magenta and blue arrows show the changes in absorption along increasing depth.

The ATR-FTIR spectra of the 13-day-aged celluloid sheets and eyeglass temple show a similar molecular decay in which the characteristic response of cellulose nitrate is still visible in the fingerprint region (Figure 11). Instead, the organ key is characterized by a greater extent of degradation, especially at the core, as the intensity of the hydroxyl stretching (νOH) increased, appearing as a broad band centered around 3400 cm^−1^. Furthermore, the disappearance of the νCOC cellulose ring band probably indicates the total collapse of the material. Concerning camphor, its infrared marker bands (marked with a black star in Figure 7) are very similar to the spectra of the eyeglass temple and 13-day sheets. The strong νC=O bands in the organ key spectra can be more likely related to the formation of the degradation products as the bands of camphor are much less recognizable.

## 4. Conclusions

This study successfully investigated the denitration of artificially aged 3D celluloid sheets at different depths and across their width via FTIR-ATR. The intensity of νC=O absorbance was not suitable to evaluate the loss of camphor because it is contemporarily influenced by the formation of carbonyl-containing degradation products. An incipient degradation gradient was detected in the moderately aged sheets even before visible changes were noticed. Furthermore, the degradation at different depths of the severely artificially aged sheets was compared to a pair of historical 3D celluloid objects. The results proved that the artificially and naturally aged specimens show the highest denitration and increase in νC=O signals, the latter being only partially explainable by the formation of degradation products in the core, most evidently at later stages of degradation. This finding also matches the gradient of degradation, which increases with depth from the surface towards the core, and which can be observed by the naked eye. The transparent celluloid sheet and eyeglass temple samples present a similar molecular extent of decay, while the opaque white organ key is characterized by a higher degree of alteration.

The artificial aging protocol applied in this study followed literature procedures [17,47,48]. Thanks to this artificial aging experiment, it was possible to conclude that studying the degradation gradient is fundamental for understanding celluloid degradation, and that sampling should consider both depth and width depending on the macroscopic appearance of alteration.

The artificial aging induced bubbling and extreme volume expansion in the core of the severely aged samples, which were not identified in naturally aged 3D museum objects so far. Nonetheless, this study can serve to plan future aging studies. From a future perspective, the measurement of the glass-transition temperature (T_g_) of celluloid would greatly support the selection of T and RH for the aging protocol. T_g_ measurement requires specific method development and data interpretation via dynamic mechanical analysis (DMA) [49,50], as the glass transition behavior is strongly dependent on the plasticizer and moisture contents. Besides exploring the influence of different intervals and combinations of T and RH, other parameters should be considered in future aging protocols, including the ventilation of the headspace (to allow or avoid volatile concentration around the sample [15]), the sample thickness (which influences the retention of degradation products [17]), the crystallinity and molecular packing that alter the porosity and permeation of celluloid at different local regions of the specimen [25], plasticizer content as well as the influence of extra compounds in the plastic mixture formulation (e.g., inorganic fillers).

The mechanisms responsible for the observed degradation gradients deserve future investigation. In particular, chromatographic techniques could be applied, following the same in-depth sampling strategy implemented in this work, to measure the molecular weight (M_w_), the nitrogen and camphor contents. In detail, size exclusion chromatography (SEC) can be used for determining the M_w_ of CN [27,51,52] and measuring the amount of camphor [27] along sample points that show distinct νC=O infrared absorptions. Ion chromatography (IC) can be applied instead to quantify N content and, in turn, to estimate the extent of denitration [17]. This multi-analytical strategy has already been implemented in a DBU project, presenting promising results [53]. ATR-FTIR can be used as a preliminary tool to investigate the degradation gradient because the proposed chromatographic methods are expensive, time-consuming, destructive and require complex sample preparation. In addition to its chemical characterization, also investigating the mechanical properties of 3D celluloid along its section would greatly support a comprehensive understanding of the degradation mechanisms and formation of alteration gradients in celluloid.

## Figures and Tables

**Table 1:** Assignment of the main bands of the ATR-FTIR spectra depicted in Figure 7.

Wavenumber (cm^−1^)			Assignment
CN Membrane (Reference)	Unaged CN Coupon	Racemic Camphor (Reference)	
3657			
	3448	3468	νO-H (bound)
2966	2963	2958	νC-H [35,36]
2923	2927	2931	ν_s_C-H [35,37]
2908			
	2887	2872	
	1728	1738	νC=O [37], from camphor [35,38,39] or CN degradation products [11,13,15,36]
1637	1635		ν_a_O-NO_2_ [35,39]
1454	1452	1447	δCH_2_ in CN [37] δ_a_CH_3_ and δ_s_CH_2_ in camphor [40]
1427	1428		δCH_2_ [35]
	1417	1416	δ_s_CH_2_ at position C3 in camphor [40]
	1391	1390	δ_s_CH_3_ in camphor [40]
1375	1374	1373	δC-H in CN [35] δ_s_CH_3_ and νC-C in camphor [40]
	1325	1324	ωCH_2_, νC-C, and δ_s_CH_3_ at C1 in camphor [40]
1278	1276	1277	ν_s_NO_2_ in CN [35,37,38,39] ωCH_2,_ νCC, with minimum ρCH_2_ and ring deformation in camphor [40]
1160	1159	1167	ν_a_O-C-C [38] νCC, ρCH_2_ and τCH_2,_ in camphor [40]
1115	1111		νCO in ring [35]
1061	1051	1045	ν_a_O-C-C attached to the NO_2_ group [38], νCOC of the cellulose ring in CN [18] νCC, τCH_2_, ρCH_2_ and in-plane δCO in camphor [40]
1022	1021	1022	νCO [37]
999	999		νC-O [37]
947	945	951	δ_s_CH [37]
918	918	914	δ_s_CH [37]
827	828	827	ν-NO in CN [37,38,39]
750	750	751	δO-NO_2_ in CN [35,37,39]
694	698		δO-NO_2_ [35,37,39]
681	676		Pyranose [37]
	554	553	
541			
	521	521	

Note: ν stretching vibration, ν_s_ symmetrical stretching vibration, ν_a_ asymmetrical stretching, δ bending vibration, δ_s_ scissoring (for CH_2_) and symmetrical deformation (for CH_3_), δ_a_ asymmetrical deformation (for CH_3_), ρ rocking, τ twisting, ω wagging.

## Data Availability

Data available on request. The data presented in this study are available on request from the corresponding author.

## Supplementary Materials

The following supporting information can be downloaded at: https://www.mdpi.com/article/10.3390/polym15030522/s1, Figure S1: Camphor molecule; Figure S2: Explanatory drawing of unaged (0 days) and artificially aged (13 days) celluloid samples with volume expansion at the center. The two layer sheets constituting the celluloid sample are highlighted. The microscopic image taken at the border of a 13-day aged sample display the interface between both.
