# Supplementary material for: Shedding Light on Degradation Gradients in Celluloid: An ATR-FTIR Study of Artificially and Naturally Aged Specimens
Source: Polymers (Basel). 2023 Jan 19;15(3):522. doi: 10.3390/polym15030522 (PMC9920386; doi:10.3390/polym15030522)
Supplement: Supplementary file 1 [file polymers-15-00522-s001.zip › polymers-2095536-supplementary.pdf]

Article

Shedding Light on Degradation Gradients in Celluloid: An ATR-FTIR Study of Artificially and Naturally Aged Specimens

Marco Valente Chavez Lozano, Christina Elsässer, Eva Mariasole Angelin and Marisa Pamplona *

Conservation Science Department, Deutsches Museum, Museumsinsel 1, 80538 Munich, Germany;
* Correspondence: m.pamplona@deutsches-museum.de

Supplementary Material

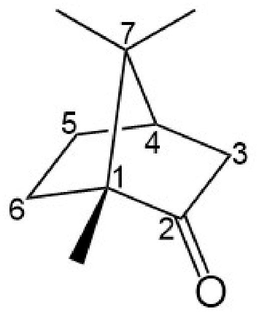

Figure S1. Camphor molecule.

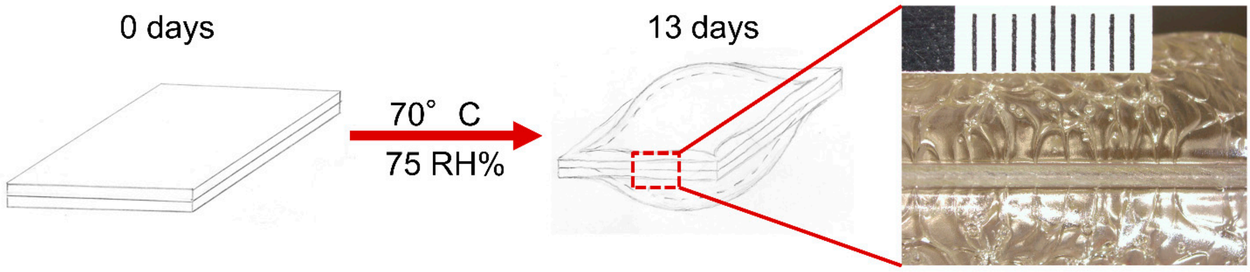

Figure S2. Explanatory drawing of unaged (0 days) and artificially aged (13 days) celluloid samples with volume expansion at the center. The two layer sheets constituting the celluloid sample are highlighted. The microscopic image taken at the border of a 13-day aged sample display the interface between both.
